# Nonapeptide Receptor Distributions in Promising Avian Models for the Neuroecology of Flocking

**DOI:** 10.3389/fnins.2018.00713

**Published:** 2018-10-16

**Authors:** Naomi R. Ondrasek, Sara M. Freeman, Karen L. Bales, Rebecca M. Calisi

**Affiliations:** ^1^Department of Neurobiology, Physiology and Behavior, University of California, Davis, Davis, CA, United States; ^2^Department of Psychology, University of California, Davis, Davis, CA, United States; ^3^California National Primate Research Center, University of California, Davis, Davis, CA, United States

**Keywords:** neuroecology, oxytocin, vasopressin, mesotocin, vasotocin, grouping behavior

## Abstract

Collective behaviors, including flocking and group vocalizing, are readily observable across a diversity of free-living avian populations, yet we know little about how neural and ecological factors interactively regulate these behaviors. Because of their involvement in mediating a variety of social behaviors, including avian flocking, nonapeptides are likely mediators of collective behaviors. To advance the neuroecological study of collective behaviors in birds, we sought to map the neuroanatomical distributions of nonapeptide receptors in three promising avian models that are found across a diversity of environments and widely ranging ecological conditions: European starlings, house sparrows, and rock doves. We performed receptor autoradiography using the commercially available nonapeptide receptor radioligands, ^125^I-ornithine vasotocin analog and ^125^I-linear vasopressin antagonist, on brain tissue sections from wild-caught individuals from each species. Because there is known pharmacological cross-reactivity between nonapeptide receptor subtypes, we also performed a novel, competitive-binding experiment to examine the composition of receptor populations. We detected binding in numerous regions throughout the brains of each species, with several similarities and differences worth noting. Specifically, we report that all three species exhibit binding in the lateral septum, a key brain area known to regulate avian flocking. In addition, sparrows and starlings show dense binding in the dorsal arcopallium, an area that has received scant attention in the study of social grouping. Furthermore, our competitive binding results suggest that receptor populations in sparrows and starlings differ in the lateral septum versus the dorsal arcopallium. By providing the first comprehensive maps of nonapeptide receptors in European starlings, house sparrows, and rock doves, our work supports the future use of these species as avian models for neuroecological studies of collective behaviors in wild birds.

## Introduction

Diverse examples of collective behaviors exist across the animal kingdom, but perhaps most conspicuous is the formation of large, coordinated groups in which individuals communicate, move, and forage together (Parrish and Edelstein-Keshet, 1999). The ecological pressures that drive or stabilize the evolution of these groups have been considered in depth (e.g., Alexander, 1974; Emlen, 1982; Solomon, 2003), but we know very little about the neural processes that prompt individuals to participate in these aggregations. Free-living birds are ideal for investigating the emergence of collective behaviors from interactions among neural systems and ecological factors—the focus of an emerging field called neuroecology (Sherry, 2006; Zimmer and Derby, 2011)—because they frequently form conspicuous groups that are comprised of individuals that feed, evade predators, and vocalize together (Helm et al., 2006). However, the neuroecology of collective behaviors has received little attention, perhaps in part because we lack well-developed organismal models suited to these types of investigations. We sought to address this gap by taking the first steps toward developing three globally distributed avian species—house sparrows (*Passer domesticus*), European starlings (*Sturnus vulgaris*), and rock doves (*Columba livia*)—as potential models for neuroecological studies of collective behaviors.

Because of their ability to invade, inhabit, and form groups in a diversity of environments, house sparrows, European starlings, and rock doves are particularly advantageous for studying how ecological variations influence the neural processes underlying collective behaviors. Since their introductions via the eastern coast of North America, these species have spread across vast swaths of the continent and today, members of each species number in the millions throughout the United States. Because of their wide distributions, these species are found across a spectrum of environmental conditions, including a variety of climates, urbanization gradients, and ecological communities (Cabe, 1993; Johnston and Janiga, 1995; Clergeau et al., 1998; Anderson, 2006). Thus, sparrows, starlings, and rock doves are ideal for intraspecies, inter-population comparisons that can reveal much about the impacts of varying ecological factors on the neurobiology underlying collective behaviors.

In addition to selecting ideal avian models, advancing the neuroecological study of collective behaviors requires that we identify candidate neural systems, ideally with demonstrated involvement in regulating social behaviors. The nonapeptide (NP) systems are an excellent place to start because they mediate a wide variety of social behaviors, including pair bonding, parent-offspring bonding, same-sex interactions, and group size preference (reviewed in Beery et al., 2016). All vertebrate species examined thus far produce NPs, a highly conserved class of neurohormones that includes oxytocin, vasopressin, and their non-mammalian homologs mesotocin and vasotocin, respectively (Gimpl and Fahrenholz, 2001; Goodson, 2005, 2013). Thus, discoveries made regarding the role of NP systems in avian collective behaviors can provide insights that support and guide similar investigations in other animal groups.

One limitation for examining NP system function in house sparrows, European starlings, and rock doves is that NP receptors have never been mapped in these species. Such maps are necessary complements to laboratory investigations, which in turn are needed to demonstrate causal links between neural and behavioral processes. In addition, studying NP systems is challenging due to a high level of structural homology and pharmacological cross-reactivity among the four subtypes of NP receptors (Acher et al., 1995; Ocampo Daza et al., 2012). This characteristic has made it difficult to identify the specific functional contributions of each receptor subtype to behavior, particularly in birds (Leung et al., 2009). Phylogenetic analyses of receptor amino acid sequences in a handful of avian species have identified four avian NP receptor subtypes (summarized in Leung et al., 2011). These studies have also shown that the two subtypes that are most highly expressed in the avian brain are vasotocin (VT) receptor 4 (VT4), which has a high degree of sequence homology to the mammalian vasopressin receptor 1a (V1aR) (Leung et al., 2011; Genbank ACCN abv24997), and avian VT3, which shares a high sequence identity with the mammalian oxytocin receptor (OTR) (Gubrij et al., 2005). Thus, our investigation focused on identifying VT4 (referred to here as V1aR-like) and VT3 (referred to here as OTR-like) as the relevant NP receptors for the current study.

To address these challenges and further the development of promising avian models for the study of the neuroecology of flocking behavior, we sought to accomplish two goals: first, to map the distribution of NP receptors in brain tissue from house sparrows, European starlings, and rock doves, and second, to identify potentially heterogenous populations of NP receptors in these species. To this end, we performed receptor autoradiography using two radioligands that are commonly employed in studies of mammalian NP receptors: ^125^I-ornithine vasotocin analog (^125^I-OVTA), which is used to label OTR, and ^125^I-linearized vasopressin antagonist (^125^I-LVA), which is used to label V1aR. We expected that ^125^I-OVTA and ^125^I-LVA would primarily label VT3 (OTR-like) and VT4 (V1aR-like), respectively. However, these radioligands produce overlapping patterns of binding in the brains of other avian species (Leung et al., 2009), which may suggest that these molecular tools bind more promiscuously to the avian NP receptors than they do in rodents. Alternatively, such overlap in radioligand binding may reflect true mixed receptor populations in specific regions of the avian brain.

To examine which specific receptor subtypes contribute to the binding patterns of each radioligand, we performed a competitive binding experiment to assess the impact that a V1aR competitor, the Manning compound, would have on ^125^I-OVTA and ^125^I-LVA binding. Due to its strong affinity for V1aR, the Manning compound is frequently used in studies of mammalian NP systems, both as a competitor to distinguish among different receptor classes for mapping purposes, and as an antagonist to examine V1aR contributions to behavioral regulation (Manning et al., 2012). We placed particular focus on determining how the Manning compound impacts ^125^I-OVTA and ^125^I-LVA binding in the lateral septum (LS) because NP receptors have been identified in this region in several avian species, and the LS has been implicated in the regulation of avian flocking behaviors (Goodson et al., 2009b; Leung et al., 2009; Kelly et al., 2011).

We selected the Manning compound for use as a putative competitor for the avian V1aR-like receptor (VT4) after first considering the molecular basis for our hypothesized pharmacological homology. In mammalian systems, the amino acids in the third and eighth positions for endogenous NPs are known to confer ligand-binding specificity by interacting with specific amino acid residues in V1aR and OTR; specifically, amino acid residues 509 and 609 in V1aR interact with the third amino acid in vasopressin (Chini et al., 1996), and residue 115 in V1aR interacts with the eighth amino acid in vasopressin (Chini et al., 1995). These three key amino acid residues in V1aR, which confer binding specificity to vasopressin, are identical in the amino acid sequence of avian VT4 (Leung et al., 2011). Additionally, the Manning compound and vasopressin are also identical in the amino acids present at the third and eighth positions (Kruszynski et al., 1980); thus, we expected that the Manning compound should bind selectively to VT4, the putative V1aR-like avian NP receptor.

Multiple studies across several avian species demonstrate that ^125^I-OVTA binds to multiple brain areas, whereas ^125^I-LVA only produces visible labeling in some, but not all, species (Goodson et al., 2006; Leung et al., 2009). We predicted that we would observe similar trends in this experiment; specifically, that ^125^I-OVTA would label NP receptors in all three of our examined species, while ^125^I-LVA would bind to receptors in only a subset of these species, across fewer brain regions, or at lower levels compared to ^125^I-OVTA. We further predicted that the Manning compound would produce more radioligand displacement in the LS when labeled receptors are V1aR-like; specifically, we expected that the Manning compound would displace ^125^I-LVA more than ^125^I-OVTA, if these radioligands are binding selectively to their corresponding avian NP receptors. Alternatively, V1aR-like and OTR-like receptors in these species may bind ^125^I-LVA, ^125^I-OVTA, and the Manning compound with similar affinities; if this is the case, we expected that the Manning compound would displace ^125^I-LVA and ^125^I-OVTA to a similar degree.

## Materials and Methods

### Animals

All birds were free-living and captured using mist nets or clap traps between 2013 and 2016. Specifically, male house sparrows (*n* = 3) were captured in November 2014 in Davis, CA, United States; female European starlings (*n* = 3) were captured in Tracy, CA, United States in January 2014; and female rock doves (*n* = 3) were captured either in Tracy, CA, United States (2 individuals) in September 2013, or in Davis, CA, United States (1 individual) in April 2016. Animal procedures were approved by the Animal Care and Use Committee of the University of California, Davis and abided by federal and state guidelines for animal care and use.

### Tissue Collection and Preparation

After capture, birds were rapidly anesthetized under isoflurane and decapitated. Brains were removed, frozen immediately on dry ice, and transferred to -80°C for storage until coronal sectioning on a cryostat. Brains were sectioned at 20 μm increments into 4 adjacent series at -20°C and subsequently mounted on to Fisher Superfrost plus slides (Fisher, Pittsburg, PA, United States), which were stored in sealed slide boxes and returned to -80°C until use for receptor autoradiography.

### Receptor Autoradiography for NP Receptors

Nonapeptide receptor autoradiography assays were carried out as previously described (Perkeybile et al., 2015; Guoynes et al., 2018; Hartman et al., 2018). Sections were allowed to thaw in slide boxes for 1 h at room temperature and then placed in racks to dry. Slides were fully submerged in 0.1% paraformaldehyde, followed by two washes in 50 mM Tris-HCl (pH 7.4). Slides were then incubated for 1 h in a solution of 50 mM Tris-HCl (pH 7.4) with 10 mM MgCl_2_, 0.1% bovine serum albumin, and 50 pM of radioligand. In this binding step, each series was then incubated in one of the following radioligand conditions: 50 pM of the OTR radioligand, ^125^I-OVTA (PerkinElmer, Boston, MA, United States) or 50 pM of the V1aR radioligand, ^125^I-LVA (PerkinElmer, Boston, MA, United States). Two of these series were incubated either in 50 pM ^125^I-OVTA plus 1 μM of the highly selective V1aR antagonist, the Manning compound, or 50 pM ^125^I-LVA plus 1 μM of the Manning compound. After the incubation period, slides were washed in multiple changes of chilled 50 mM Tris-HCl (pH 7.4) with 10 mM MgCl_2_. Slides were then placed in a final rinse of this solution for 30 min, with gentle stirring, then rinsed in ddH_2_O and allowed to air-dry overnight. Slides were then apposed to Carestream BioMax MR film (Kodak, Rochester, NY, United States) with a set of ten ^125^I microscale standards (American Radiolabeled Chemicals, Inc., St. Louis, MO, United States) for 4 days, then developed and analyzed.

### Imaging and Quantification

Photography of autoradiography films and quantification of regions with visible binding above background were accomplished using the MCID Digital Densitometry Core System (Interfocus Imaging, Cambridge, United Kingdom). Optical binding density (OBD) was quantified by extrapolation from a standard curve, which was constructed using a set of autoradiography standards (American Radiolabeled Chemicals, Inc., St. Louis, MO, United States) that were apposed to film in conjunction with specimen slides. For each bird, specific binding values for areas with visible binding were averaged across three sections for each area of interest. To account for individual differences in non-specific binding, OBD was measured in each section in a background area where no visible binding was apparent. For each section, specific binding was calculated by subtracting the non-specific binding value from OBD values obtained for each area. For the competitive binding experiment, labeling in the LS was quantified across all three species, and labeling in the dorsal arcopallium (Ad) was quantified in house sparrows and starlings, but not in rock doves due to a lack of ^125^I-OVTA and ^125^I-LVA binding in this area.

Identification of labeled brain regions was accomplished by referencing avian brain atlases and key neuroanatomical landmarks, visible on slides and in photomicrographs. Brain regions were identified in house sparrows using Nixdorf-Bergweiler and Bischof (2007), in European starlings using Nixdorf-Bergweiler and Bischof (2007) and De Groof et al. (2016), and in rock doves using Karten and Hodos (1967). Names for brain regions identified using Karten and Hodos (1967) were updated according to Reiner et al. (2004) and Jarvis et al. (2005).

### Statistical Analysis

Because work in several songbird species implicates the LS in the regulation of flocking behavior, comparisons of OBD values across binding conditions were planned *a priori* and used two-tailed *t*-tests or Wilcoxon rank sum tests. To minimize the risk of inflating the type 1 error rate, only a subset of all possible comparisons was performed. These planned comparisons excluded only those that present little heuristic value, an approach that has been described in detail elsewhere (Ruxton and Beauchamp, 2008; Ondrasek et al., 2015). Specifically, the comparisons were as follows: ^125^I-OVTA versus ^125^I-LVA, to identify differences in binding density for these two ligands; ^125^I-OVTA versus ^125^I-OVTA + Manning compound, to examine the impacts of the competitor on ^125^I-OVTA binding; ^125^I-LVA versus ^125^I-LVA + Manning compound, to assess the impacts of the competitor on ^125^I-LVA binding; and ^125^I-LVA + Manning compound versus ^125^I-OVTA + Manning compound, to determine if the competitor had differential impacts on ^125^I-LVA versus ^125^I-OVTA binding. The decision to use t-tests assuming equal or unequal variances was made subsequent to Bartlett’s test for unequal variance.

In starlings and house sparrows, ^125^I-LVA and ^125^I-OVTA binding densities were particularly high in Ad. Because dense ^125^I-OVTA labeling in the arcopallium has been reported in several other songbird species (Leung et al., 2009; Wilson et al., 2016), and because of this area’s putative homology to the mammalian amygdala—a region with significant contributions to social behavior in mammals (Jarvis, 2009; Hanics et al., 2016)—*post hoc* comparisons of OBD values across binding conditions were performed using the Steel-Dwass method for non-parametric multiple comparisons, following ANOVA.

To provide a further test of differences in ^125^I-OVTA and ^125^I-LVA binding, and to identify general species effects on radioligand binding patterns, we combined ^125^I-OVTA and ^125^I-LVA optical binding densities for all three species and performed a principal component (PC) analysis. Only the 32 brain areas showing either ^125^I-OVTA or ^125^I-LVA binding in at least one species were included in the analysis. PC scores were subsequently analyzed using ANOVAs and non-parametric tests. Additional details regarding these analyses—including PC loadings, statistical test outcomes, and an interpretation of the results— may be found in the **Supplementary Materials**.

All statistical analyses were completed using JMP Pro 12 (SAS Institute Inc., Cary, NC, United States). Means ± SEM are reported throughout, and all OBD values included in statistical analyses and reported in figures and tables have been corrected for non-specific binding as described above.

## Results

### General Observations

We expected that ^125^I-OVTA and ^125^I-LVA would primarily label the avian OTR-like receptor (VT3) and the avian V1aR-like receptor (VT4), respectively. Based on analyses of homologous amino acid sequences, we also hypothesized that the Manning compound should bind selectively to V1aR-like receptors in avian brains, as it does in mammals. Binding of ^125^I-OVTA and ^125^I-LVA was widely dispersed across a variety of brain regions in European starlings and rock doves, but comparatively more restricted in house sparrows (for complete lists and abbreviations of regions showing binding, see **Tables 1**–**4**). Save one exception in starling brain, ^125^I-LVA binding always occurred in areas that also showed ^125^I-OVTA binding, but the reverse was not always true. For instance, in rock doves, ^125^I-OVTA, but not ^125^I-LVA, signal was apparent in arcopallium (A), basorostral pallial nucleus (Bas), entopallium (E), and mesopallium (M). Of the two radioligands, ^125^I-OVTA binding produced a more intense signal in most areas, whereas incubation with ^125^I-LVA resulted in far more non-specific binding (i.e., unilateral binding, often without distinct shape or edges, that was not repeated across two or more sections). In addition, intraspecies variation in ^125^I-OVTA and ^125^I-LVA binding was apparent in numerous brain areas across all three species; for many regions, binding was observed in some, but not all individuals (**Tables 2**–**4**).

**Table 1:** Abbreviations for avian brain areas.

Abbreviation	Brain region
A	Arcopallium
Ad	Dorsal arcopallium
Al	Lateral arcopallium
APH	Parahippocampal area
Bas	Basorostral pallial nucleus
Cb	Cerebellum
CcS	Caudocentral septum
CMM	Caudomedial mesopallium
CoS	Commissural septal nucleus
H	Hyperpallium
LMAN	Lateral magnocellular nucleus of the anterior nidopallium
LS	Lateral septum
M	Mesopallium
MBH	Mediobasal hypothalamus
MMAN	Medial magnocellular nucleus of the anterior nidopallium
MSt	Medial striatum
N	Nidopallium
NCM	Caudal medial nidopallium
OMd	Dorsal nucleus of the oculomotor nerve
OMv	Ventral nucleus of the oculomotor nerve
Ov	Ovoid nucleus
pHVC	Para-high vocal center
RA	Robust nucleus of the arcopallium
TeO	Optic tectum
TnA	Nucleus taeniae of the amygdala
Uva	Uvaeform nucleus
VMH	Ventromedial hypothalamus
DLP	Dorsolateral nucleus of the posterior thalamus
DMP	Dorsomedial nucleus of the posterior thalamus
E	Entopallium
Hp	Hippocampus
Lhy	Lateral hypothalamus
MVL	Ventrolateral nucleus of the mesopallium
NIM	Intermediate medial nidopallium
SGP	Periventricular gray and fibrous tectal layers


**Table 2:** Mean optical binding density (±SEM) of ^125^I-OVTA and ^125^I-LVA in brain regions with visible binding in house sparrow.

Brain region	^125^I-OVTA	^125^I-LVA
Ad	3433 ± 658 (ABC)	2956 ± 195 (AB)
APH	1153 ± 221 (ABC)	538 ± 92 (BC)
CMM	482 ± 28 (B)	245 ± 23 (B)
H	429 ± 71 (B)	360 ± 41 (C)
LS	1087 ± 67 (ABC)	158 ± 157 (C)
MMAN	732 ± 158 (ABC)	369 ± 120 (B)
MSt	240 ± 39 (C)	693 ± 84 (AC)
OMd	911 ± 114 (B)	651 ± 54 (B)
OMv	793 ± 19 (B)	797 ± 107 (B)
pHVC	1290 ± 232 (AB)	815 ± 102 (B)
TeO	571 ± 55 (ABC)	636 ± 81 (ABC)


**Letters (A, B, or C) represent individual birds that showed binding in the indicated brain area.**

**Table 3:** Mean optical binding density (±SEM) of ^125^I-OVTA and ^125^I-LVA in brain regions with visible binding in European starling.

Brain region	^125^I-OVTA	^125^I-LVA
Ad	2685 ± 85 (ABC)	1481 ± 70 (ABC)
Al	3976 ± 186 (ABC)	2587 ± 269 (ABC)
APH	968 ± 117 (ABC)	627 ± 93 (ABC)
Bas	1595 ± 45 (C)	–
CcS	1070 ± 168 (ABC)	633 ± 111 (BC)
CMM	–	630 ± 32 (C)
CoS	2190 ± 150 (AC)	–
H	768 ± 127 (ABC)	765 ± 215 (C)
LMAN	981 ± 139 (ABC)	–
LS	4234 ± 490 (ABC)	1480 ± 456 (ABC)
M	983 ± 122 (ABC)	841 ± 107 (BC)
MBH	595 ± 233 (B)	–
MSt	591 ± 91 (B)	–
N	1026 ± 140 (ABC)	855 ± 182 (BC)
NCM	2008 ± 250 (ABC)	931 ± 103 (ABC)
OMd	521 ± 84 (A)	–
OMv	449 ± 10 (A)	–
pHVC	3539 ± 479 (ABC)	1826 ± 350 (BC)
Uva	1523 ± 132 (ABC)	627 ± 48 (ABC)
RA	1756 ± 83 (ABC)	805 ± 122 (ABC)
TeO	792 ± 50 (ABC)	585 ± 118 (ABC)
TnA	1045 ± 169 (ABC)	998 ± 268 (ABC)
VMH	935 ± 210 (ABC)	914 ± 469 (B)


**Letters (A, B, or C) represent individual birds that showed binding in the indicated brain area (–, area not distinguishable from background binding)*.*

**Table 4:** Mean optical binding density (±SEM) of ^125^I-OVTA and ^125^I-LVA in brain regions with visible binding in rock dove.

Brain region	^125^I-OVTA	^125^I-LVA
A	662 ± 100 (BC)	–
APH	983 ± 223 (ABC)	765 ± 145 (BC)
Bas	1534 ± 364 (ABC)	–
CMM	801 ± 196 (ABC)	743 ± 149 (ABC)
DLP	1628 ± 73 (ABC)	1228 ± 97 (BC)
DMP	793 ± 46 (BC)	–
E	671 ± 75 (ABC)	–
H	540 ± 54 (BC)	–
Hp	1084 ± 97 (BC)	643 ± 117 (B)
LHy	411 ± 57 (BC)	–
LS	2692 ± 221 (ABC)	936 ± 221 (ABC)
M	612 ± 92 (BC)	–
MSt	3613 ± 235 (C)	890 ± 188 (C)
MVL	1493 ± 145 (BC)	483 ± 57 (B)
NIM	1596 ± 156 (BC)	1270 ± 397 (C)
SGP	576 ± 117 (ABC)	–
TeO	312 ± 36 (ABC)	542 ± 108 (B)


**Letters (A, B, or C) represent individual birds that showed binding in the indicated brain area (–, area not distinguishable from background binding).**

### Distribution of ^125^I-OVTA and ^125^I-LVA Binding in House Sparrow

**FIGURE 1:**
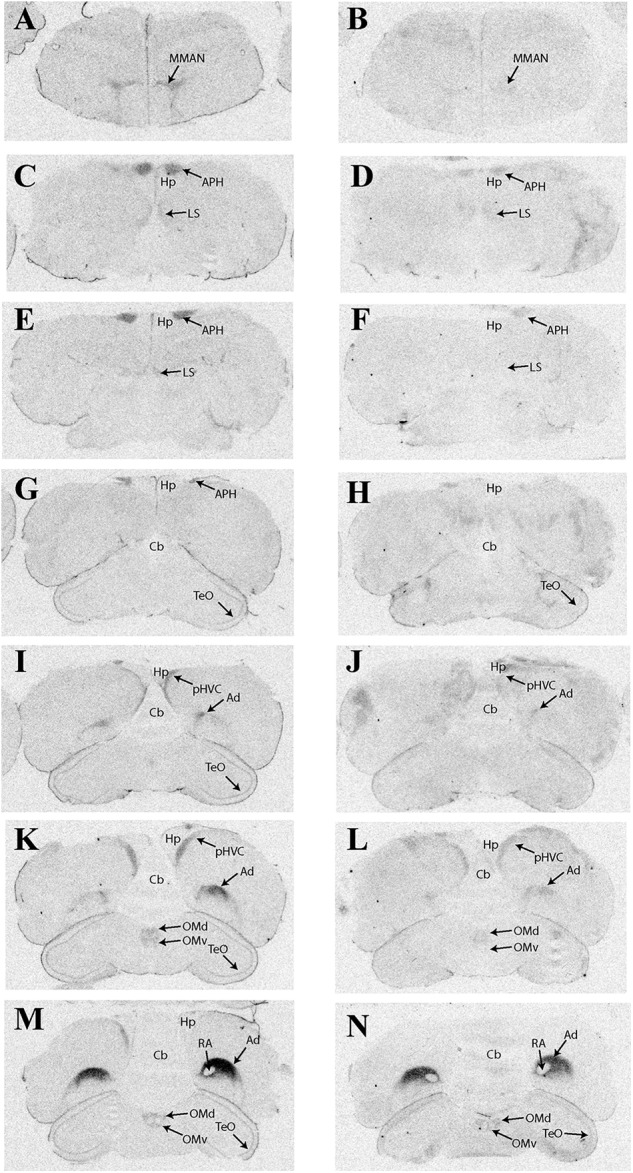
Representative photomicrographs of ^125^I-ornithine vasotocin analog (^125^I-OVTA; **A,C,E,G,I,K,M**) or ^125^I-linearized vasopressin antagonist (^125^I-LVA; **B,D,F,H,J,L,N**) binding in the brain of a house sparrow (images correspond to individual “B” in **Table 2**).

**Table 2** provides a complete list of brain regions that presented with visible radioligand binding, and **Figure 1** shows representative autoradiograms of ^125^I-OVTA and ^125^I-LVA binding sites in house sparrows. ^125^I-OVTA and ^125^I-LVA binding was limited to relatively few brain regions, although labeling was widely dispersed across the rostral-caudal axis of the brain. Unlike in European starlings and rock doves, in which the highest level of binding was observed in portions of the septal complex, in house sparrows the densest ^125^I-OVTA signal was observed in the Ad, a trend that was noted across all three individuals. Other areas showing dense ^125^I-OVTA binding include the parahippocampal area (APH), the LS, and the para-high vocal center (pHVC). Binding in the LS and APH occurred across all three individuals, while pHVC labeling was observed in two out of three subjects. ^125^I-LVA binding occurred only in sites that also showed ^125^I-OVTA binding. In addition, the presence of ^125^I-LVA binding was highly variable across individuals, such that ^125^I-LVA signal occurred in some, but not all individuals for all regions except for the optic tectum (TeO).

### Distribution of ^125^I-OVTA and ^125^I-LVA Binding in European Starling

**FIGURE 2:**
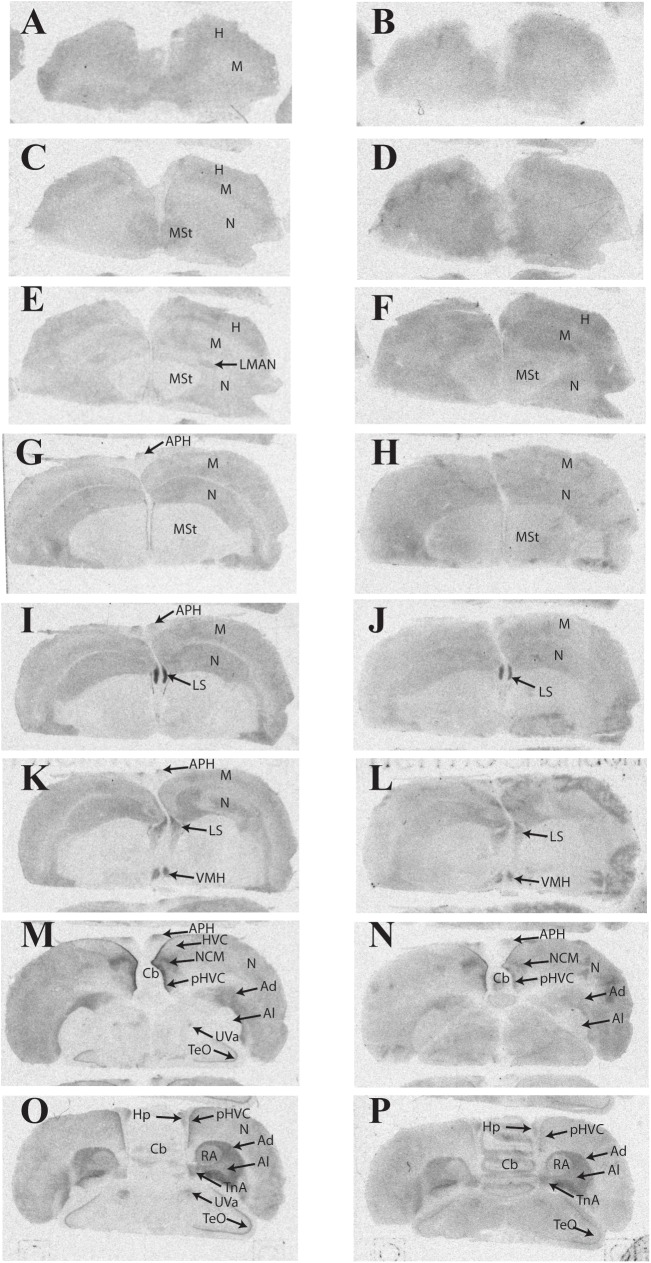
Representative photomicrographs of ^125^I-ornithine vasotocin analog (^125^I-OVTA; **A,C,E,G,I,K,M,O**) or ^125^I-linearized vasopressin antagonist (^125^I-LVA; **B,D,F,H,J,L,N,P**) binding in the brain of a European starling (images correspond to individual “B” in **Table 3**).

Representative autoradiograms and a complete list of brain areas in the European starling with visible binding appear in **Figure 2** and **Table 3**, respectively. All three female starlings showed high levels of ^125^I-OVTA binding in the LS, Ad, lateral arcopallium (Al), and pHVC, with the strongest signals occurring in the LS. More moderate ^125^I-OVTA binding occurred across all three females in portions of the nidopallium, especially the caudal medial region (NCM); the commissural septal nucleus (CoS); the uvaeform nucleus (Uva); robust nucleus of the arcopallium (RA); and the nucleus taeniae of the amygdala (TnA). As in house sparrows, ^125^I-LVA binding almost exclusively overlapped with ^125^I-OVTA binding sites, with the exception of the caudomedial mesopallium (CMM), which showed ^125^I-LVA signal in one female. Similar to ^125^I-OVTA, the highest density of ^125^I-LVA binding sites occurred in the LS, Ad, Al, and pHVC, although binding did not appear in all three subjects for all of these regions. In comparison to ^125^I-OVTA, the distribution of ^125^I-LVA binding sites was more limited and, in all cases in which both radioligands bound to a region, ^125^I-LVA binding density was lower.

### Distribution of ^125^I-OVTA and ^125^I-LVA Binding in Rock Dove

**FIGURE 3:**
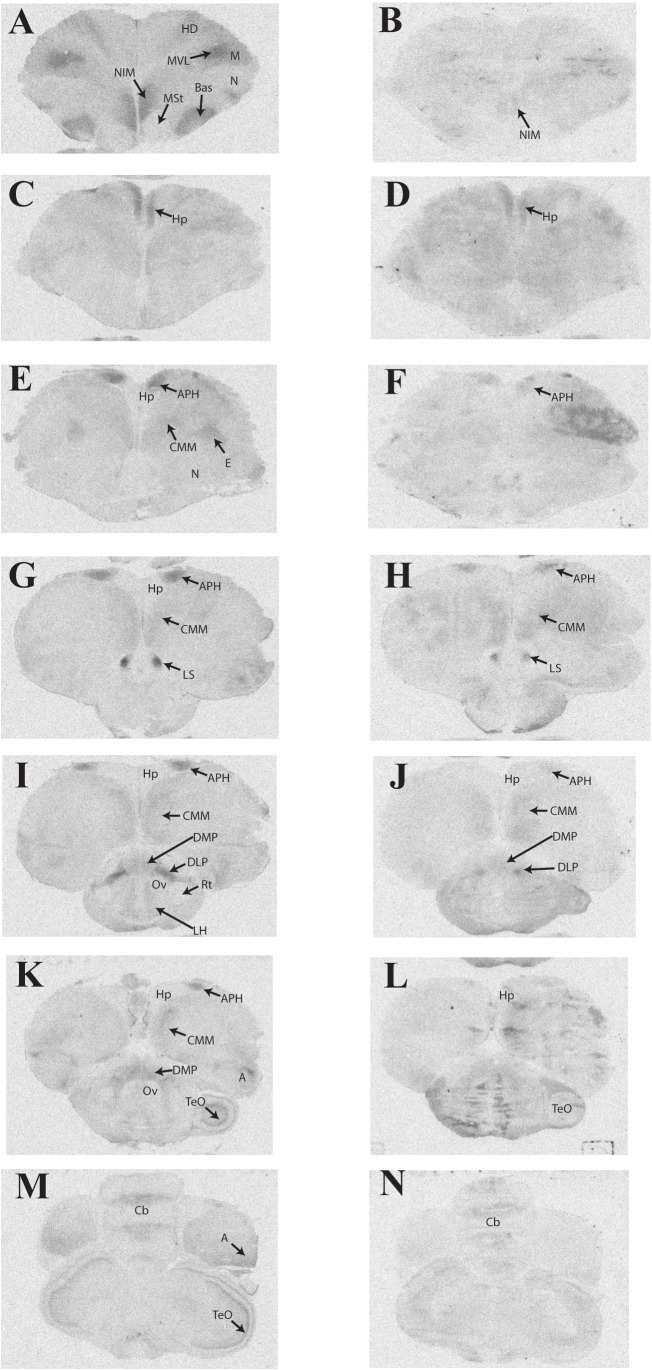
Representative photomicrographs of ^125^I-ornithine vasotocin analog (^125^I-OVTA; **A,C,E,G,I,K,M**) or ^125^I-linearized vasopressin antagonist (^125^I-LVA; **B,D,F,H,J,L,N**) binding in the brain of a rock dove (images correspond to individual “B” in **Table 4**).

**FIGURE 4:**
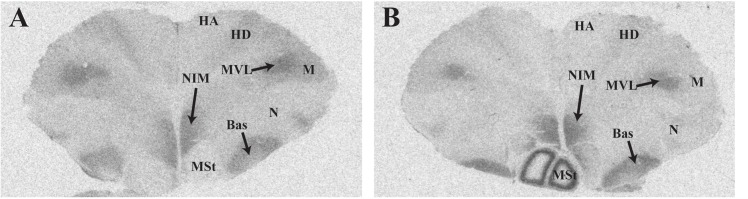
Photomicrographs showing diverse binding patterns of ^125^I-ornithine vasotocin analog (^125^I-OVTA) in medial striatum of two female rock doves. For the bird represented by the right panel **(B)**, incubation with ^125^I-linearized vasopressin antagonist (^125^I-LVA) produced similar ring-like binding in the medial striatum, although the signal was less intense. Images in the left **(A)** and right **(B)** panels correspond with individuals “B” and “C,” respectively, in **Table 4**. HA, apical hyperpallium; HD, densicellular hyperpallium.

Representative photomicrographs and a complete list of brain regions showing radioligand binding in rock doves appear in **Figure 3** and **Table 4**, respectively. As in European starlings, ^125^I-OVTA binding was more broadly distributed and denser than ^125^I-LVA binding. High levels of ^125^I-OVTA labeling were noted in the medial striatum (MSt), basorostral pallial nucleus (Bas), dorsolateral nucleus of the posterior thalamus (DLP), hippocampus (Hp), LS ventrolateral nucleus of the mesopallium (MVL), and the intermediate medial nidopallium (NIM). Binding in several of these regions—MSt, Hp, MVL, and NIM—was only apparent in a subset of females. ^125^I-LVA binding was highest in the NIM and DLP, though labeling in these regions appeared in fewer subjects compared to ^125^I-OVTA. This trend—observing ^125^I-LVA labeling in fewer subjects compared to ^125^I-OVTA—was repeated across all brain areas except the CMM, LS, and MSt. Regarding the latter, ^125^I-LVA and ^125^I-OVTA binding occurred in a distinct ring-like pattern in one female, whereas the other two subjects showed no observable labeling in MSt (**Figure 4**).

### Competitive Binding Patterns in Lateral Septum of House Sparrows, European Starlings, and Rock Doves

**FIGURE 5:**
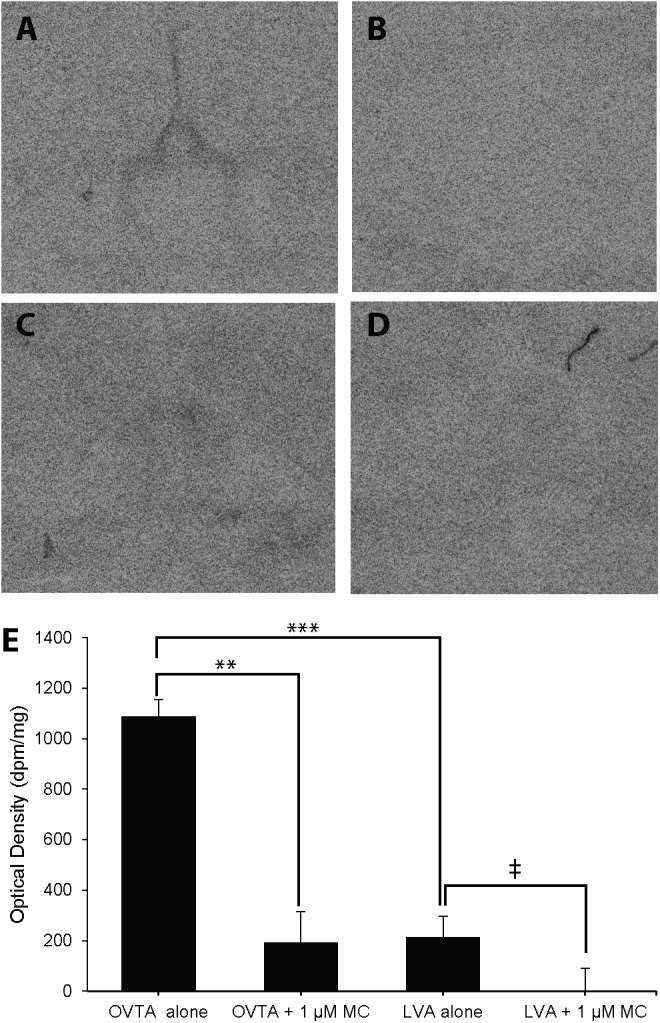
Effects of a competitor, Manning Compound (MC) on mean optical binding density (+SEM) for ^125^I-OVTA and ^125^I-LVA in the lateral septum of a house sparrow (**A**, ^125^I-OVTA alone; **B**, ^125^I-OVTA plus MC; **C**, ^125^I-LVA alone; **D**, ^125^I-LVA plus MC). **(A–D)** Correspond to individual “C” in **Table 2**. Symbols above brackets in the chart **(E)** indicate significant and near significant differences between binding conditions (^∗∗∗^*P* < 0.001, ^∗∗^*P* < 0.01, ^‡^*P* = 0.05).

**FIGURE 6:**
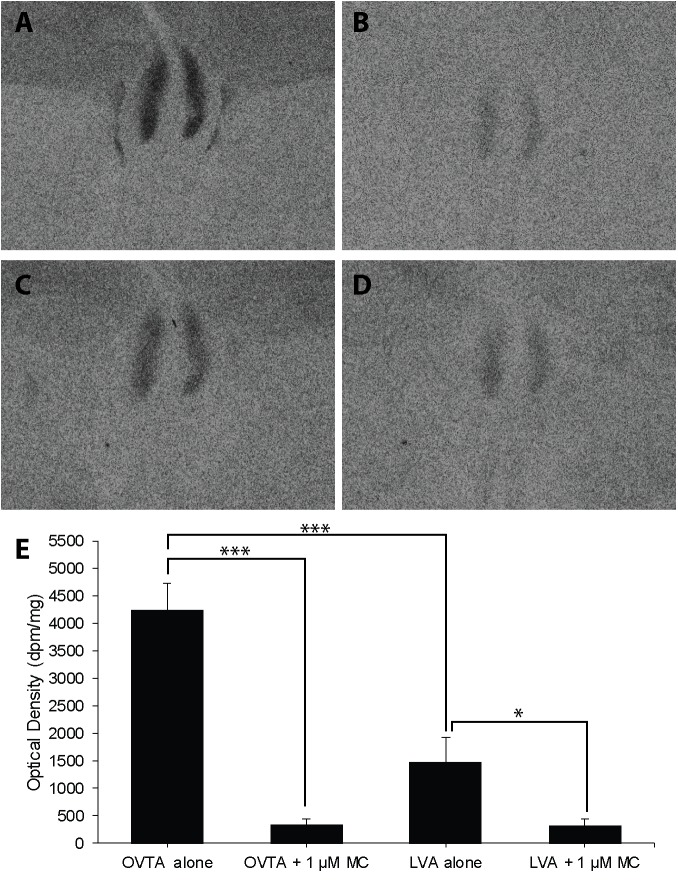
Effects of a competitor, Manning Compound (MC) on mean optical binding density (+SEM) for^125^I-OVTA and ^125^I-LVA in the lateral septum of a European starling (**A**, ^125^I-OVTA alone; **B**, ^125^I-OVTA plus MC; **C**, ^125^I-LVA alone; **D**, ^125^I-LVA plus MC). **(A–D)** Correspond to individual “B” in **Table 3**. Asterisks above brackets in the chart **(E)** indicate significant differences between binding conditions (^∗∗∗^*P* < 0.001, ^∗^*P* < 0.05).

**FIGURE 7:**
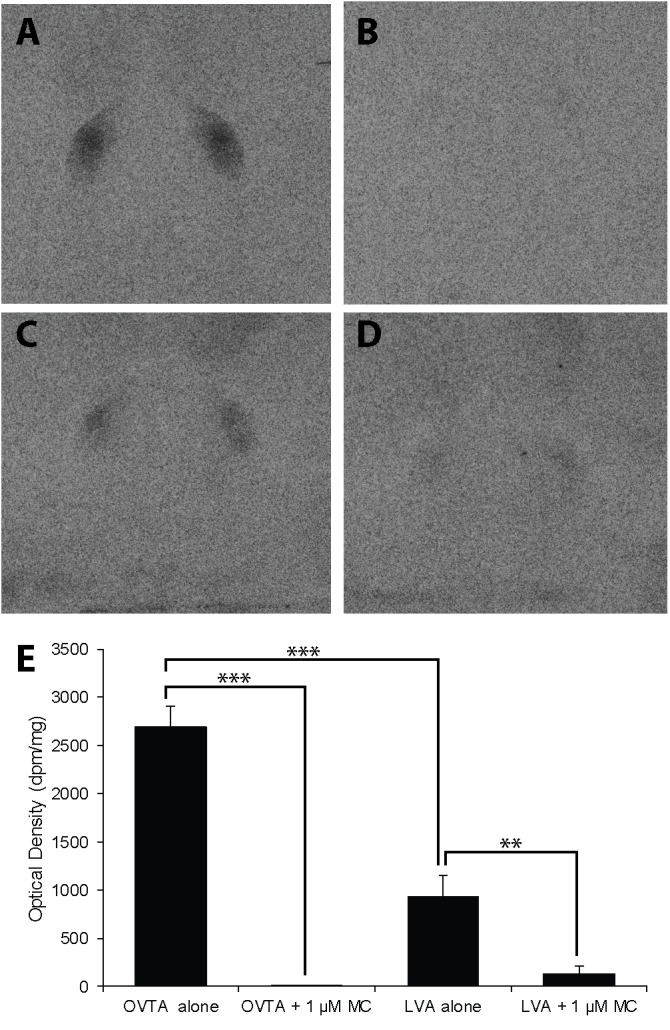
Effects of a competitor, MC on mean optical binding density (+SEM) for^125^I-OVTA and ^125^I-LVA in the lateral septum of a rock dove (**A**, ^125^I-OVTA alone; **B**, ^125^I-OVTA plus MC; **C**, ^125^I-LVA alone; **D**, ^125^I-LVA plus MC). **(A–D)** Correspond to individual “C” in **Table 4**. Asterisks above brackets in the chart **(E)** indicate significant differences between binding conditions (^∗∗∗^*P* < 0.001, ^∗∗^*P* < 0.01).

The impacts of the Manning compound on binding patterns in the LS were strikingly consistent across all three species (house sparrows, **Figure 5**; European starlings, **Figure 6**; rock doves, **Figure 7**). In rock doves and European starlings, the competitor significantly reduced binding of both ^125^I-OVTA [rock doves: *Z* = 3.60, *P* = 0.0003; European starlings: *t*(8.67) = 7.77, *P* < 0.0001] and ^125^I-LVA [rock doves: *t*(10.07) = 3.41, *P* = 0.007; European starlings: *t*(9.13) = 2.43, *P* = 0.04]. Similar trends were observed in house sparrows, where the Manning compound induced significant and near significant reductions in binding for ^125^I-OVTA (*Z* = 3.24, *P* = 0.001) and ^125^I-LVA (*Z* = 1.94, *P* = 0.05), respectively. In all three species, ^125^I-OVTA binding was significantly higher than ^125^I-LVA in the absence of the Manning compound [rock doves: *t*(16) = 5.62, *P* < 0.0001; European starlings: *t*(16) = 4.12, *P* = 0.0008; house sparrows: *t*(16) = 8.22, *P* < 0.0001], but addition of the competitor eliminated this difference.

### Competitive Binding Patterns in Dorsal Arcopallium of House Sparrows and European Starlings

**FIGURE 8:**
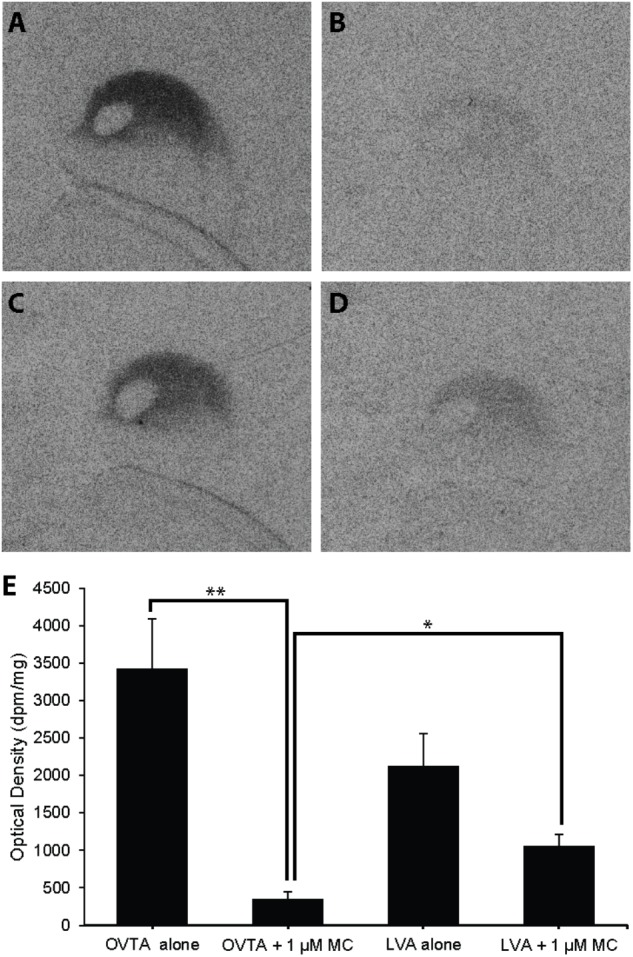
Effects of a competitor, MC on mean optical binding density (+SEM) for^125^I-OVTA and ^125^I-LVA in the arcopallium of a house sparrow (**A**, ^125^I-OVTA alone; **B**, ^125^I-OVTA plus MC; **C**, ^125^I-LVA alone; **D**, ^125^I-LVA plus MC). **(A–D)** Correspond to individual “B” in **Table 2**. Asterisks above brackets in the chart **(E)** indicate significant differences between binding conditions (^∗∗^*P* < 0.01, ^∗^*P* < 0.05).

**FIGURE 9:**
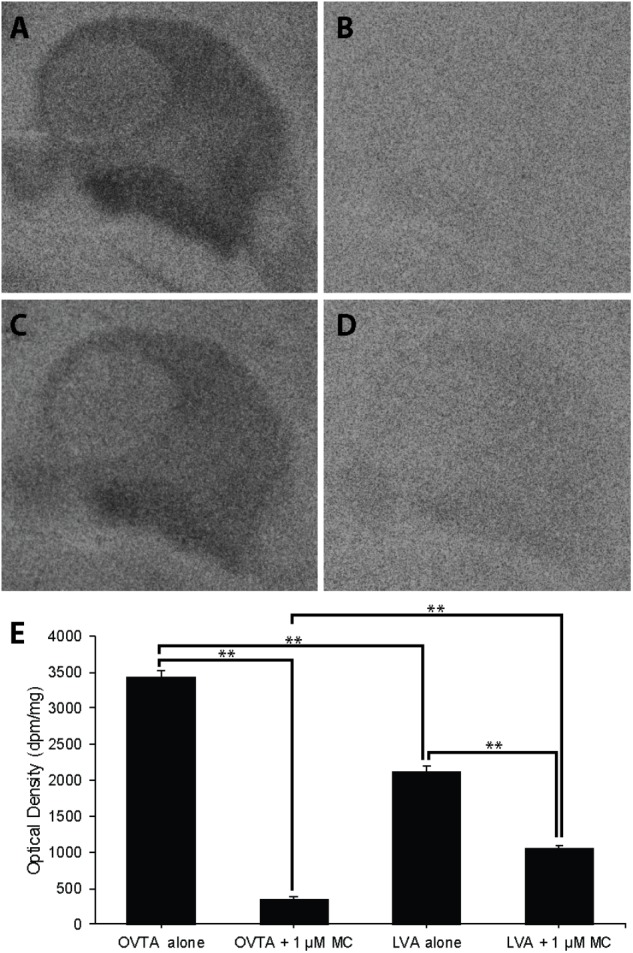
Effects of a competitor, MC on mean optical binding density (+SEM) for ^125^I-OVTA and ^125^I-LVA in the arcopallium of a European starling (**A**, ^125^I-OVTA alone; **B**, ^125^I-OVTA plus MC; **C**, ^125^I-LVA alone; **D**, ^125^I-LVA plus MC). **(A–D)** Correspond to individual “B” in **Table 3**. Asterisks above brackets in the chart **(E)** indicate significant differences between binding conditions (^∗∗^*P* < 0.01).

Competitive binding patterns were similar across house sparrows (**Figure 8**) and European starlings (**Figure 9**). In the absence of the Manning compound in both sparrows and starlings, ^125^I-OVTA binding in Ad was higher than ^125^I-LVA, though this effect was significant for starlings (*Z* = 3.53, *P* = 0.002), but not sparrows (*Z* = 1.59, *P* = 0.38). Addition of the competitor significantly decreased ^125^I-OVTA binding in both sparrows (*Z* = 3.54, *P* = 0.002) and starlings (*Z* = 3.53, *P* = 0.002). Addition of the Manning compound similarly reduced ^125^I-LVA binding, though this effect was significant in European starlings (*Z* = 3.53, *P* = 0.002), but not house sparrows (*Z* = 1.50, *P* = 0.44). Although the Manning compound reduced binding of both radioligands, ^125^I-LVA binding was significantly higher than ^125^I-OVTA in the presence of the competitor, a trend that was observed in both sparrows (*Z* = 2.65, *P* = 0.04) and starlings (*Z* = 3.53, *P* = 0.002). In rock doves, two of three females showed low ^125^I-OVTA binding in the arcopallium; ^125^I-LVA binding was absent in this region. Thus, competitive binding patterns were not assessed in the arcopallium in rock doves.

## Discussion

The goals of our research were twofold: first, to establish neuroanatomical maps of NP receptors in three promising models for neuroecological examinations of collective behavior, and second, to examine the composition of NP receptor populations of these species using competitive binding. Our findings confirm our prediction that ^125^I-LVA binding would be more limited than ^125^I-OVTA binding and support the existence of multiple NP receptor types with overlapping distributions. Below, we discuss binding patterns in rock doves, European starlings, and house sparrows in the context of NP receptor maps reported for other avian species; discuss the functional implications of binding in specific brain areas; and discuss the implications of our work for future neuroecological investigations of grouping behaviors. Although this study was not designed to provide a robust quantitative test of interspecies differences in NP receptor density or distribution, qualitative examination of our results highlights potentially valuable, novel lines of inquiry for understanding the neuroecological bases of collective behaviors, which we discuss further below.

### Radioligand Binding in an Interspecies Context

Similar to reports in other avian species (Goodson et al., 2006; Leung et al., 2009), we found that ^125^I-LVA binding was limited across all three species. Specifically, we found that ^125^I-LVA signal appeared in fewer brain regions, in fewer individuals, and at lower densities when compared to ^125^I-OVTA. In house sparrows and European starlings, the most pronounced ^125^I-LVA binding occurred in portions of the arcopallium, while in rock doves, the highest level of ^125^I-LVA signal appeared in the DLP and NIM, though only a subset of individuals showed binding in these regions. All three species showed ^125^I-LVA binding in LS. These results replicate similar findings of limited ^125^I-LVA binding, often restricted to LS, in other avian species. For example, among several flocking and territorial Estrildid finch species [melba finch (*Pytilia melba*), violet-eared waxbill (*Uraeginthus granatina*), Angolan blue waxbill (*Uraeginthus angolensis*), spice finch (*Lonchura punctulata*), and zebra finch (*Taeniopygia guttata*)], only the spice finch shows pronounced binding outside of the LS (Goodson et al., 2006). Similarly, in the white-throated sparrow (*Zonotrichia albicollis*), ^125^I-LVA binding is restricted to the septal nuclei, Ad, and TeO (Leung et al., 2009). Although these avian taxa show similar ^125^I-LVA binding patterns, they display varying degrees of grouping behavior, suggesting some degree of evolutionary conservation in brain-wide distribution for the receptor, or receptors, to which ^125^I-LVA binds. However, variations in NP receptor distribution or density within specific brain regions may contribute to behavioral differences. For example, localized ^125^I-LVA binding *within* septal areas has been associated with differences in grouping behavior among flocking and territorial avian species; similar findings have also been reported for ^125^I-OVTA (Goodson et al., 2006, 2009b).

In contrast to ^125^I-LVA, ^125^I-OVTA binding was more intense and widely distributed in all three species. In house sparrows and European starlings, moderate to high levels of ^125^I-OVTA binding occurred in the arcopallium, APH, septal areas, and pHVC. European starlings showed dense ^125^I-OVTA binding in additional brain areas, including the NCM and TnA. The distribution of ^125^I-OVTA binding in European starlings and house sparrows showed a number of similarities to ^125^I-OVTA binding patterns in other songbird species. For example, in the white-throated sparrow and zebra finch, ^125^I-OVTA binds to receptors in the LS, TnA, APH, and arcopallium (Leung et al., 2009), and in several species of emberizid sparrow (field sparrow (*Spizella pusilla*), dark-eyed junco (*Junco hyemalis*), song sparrow (*Melospiza melodia*), and eastern towhee (*Pipilo erythrophthalamus*), ^125^I-OVTA binds to the LS and arcopallium (Wilson et al., 2016). As in European starlings and house sparrows, ^125^I-OVTA binding in rock doves was high in LS, but the overall ^125^I-OVTA binding pattern showed several distinctions in rock doves relative to the other two species. Specifically, in rock doves, high ^125^I-OVTA binding appeared in Bas, DLP, Hp, MVL, and NIM, but not in the arcopallium. In addition, one rock dove showed a striking and, to our knowledge, previously unreported distribution of NP receptors in a ring-like pattern along the MSt’s outer margins.

The distinct binding patterns in the brain of the rock dove, when compared to European starlings and house sparrows, may have a variety of underlying causes, including evolutionarily driven interspecies differences, differences in the season of specimen collection, or differences in natural history or life history stage. Regarding the first explanation, it is worth noting that European starlings and house sparrows are both songbirds and more evolutionarily related to one another than to rock doves, which is a Columbiforme (an order of birds that includes pigeons and doves; Johnston and Janiga, 1995). Because our study was not designed to elucidate interspecies differences in NP receptor maps, future work will be needed to examine the validity of these explanations. Approximately half of all extant avian species are *not* songbirds (Barker et al., 2004); however, thus far all studies examining the relationship between NPs and grouping behavior in birds have used songbird species. Comparisons across both songbird and non-songbird taxa are needed to augment our understanding of the neural mechanisms that underlie flocking, as well as the generality of these mechanisms across avian species.

### Grouping Behavior and NP Receptors in the Lateral Septum

In both mammals and birds, the LS appears to play an important role in regulating intra- and interspecies differences in social behavior. For example, female meadow voles (*Microtus pennsylvanicus*), which form groups in winter, show variations in same-sex huddling that are associated with OTR expression in the LS (Beery and Zucker, 2010). Similarly, social (*Ctenomys sociabilis*) and solitary (*C. haigi*) species of rodents known as tuco–tucos show differences in OTR binding in LS (Beery et al., 2008a). In the zebra finch, NP receptors in the septal complex are associated with variations in group size preference (Goodson et al., 2009b). In addition, interspecies comparisons of estrildid finches show that ^125^I-LVA and ^125^I-OVTA binding in the caudal zone of the LS is higher in flocking versus territorial species, and infusions of V1aR and OTR antagonists directly into the zebra finch LS significantly decrease the duration of time that individuals spend near a large group of conspecifics (Goodson et al., 2006, 2009b; Kelly et al., 2011). Intriguingly, variations in mesotocin innervation, but not NP receptor densities, in the LS are associated with different seasonal patterns of flocking behavior (i.e., flocking year-round versus winter flocking) across species of emberizid sparrows (Goodson et al., 2012; Wilson et al., 2016). These findings suggest that interspecies and seasonal variations in flocking may be differentially mediated by the NP systems, and highlight the importance of avoiding the assumption that a single mechanism governs apparently similar behavioral patterns. They also support consideration of brain areas other than the LS as potential mediators of seasonal variations in flocking.

### Brain Areas With Unknown Contributions to Flocking: The Arcopallium and Sensory Pathways

Much focus has been placed on the LS in studies of NPs and their role in avian grouping behavior; however, several brain areas other than the LS also show dense expression of NP receptors. For example, using multiple songbird species, Leung et al. (2009) and Wilson et al. (2016) found moderate to high concentrations of NP receptors in the arcopallium and the caudal nidopallium, an area involved in auditory perception. Similarly, we found dense NP receptor expression in the arcopallium, particularly in the dorsal zone (in house sparrows and European starlings, but not rock doves), and in the caudomedial nidopallium (NCM; in European starlings). Little is known about the function of NP receptors in these brain regions, although there is reason to suspect that they may be involved in mediating social behavior. For example, Wilson et al. (2016) identified the rostral arcopallium as a potential “affiliation hot spot” in the avian brain because of its putative homology to the mammalian pallial amygdala, a region with well-established contributions to social behavior (Jarvis, 2009; Hanics et al., 2016). Furthermore, Wilson et al. (2016) found that seasonally flocking, but not non-flocking species of emberizid sparrows, show higher ^125^I-OVTA binding in the rostral arcopallium during winter. In combination with our results, these findings implicate NPs in the arcopallium as potential mediators of seasonal variations in flocking, and support future investigations of this possibility in European starlings and house sparrows, but not in rock doves, which did not show robust radioligand binding in the arcopallium.

Although NP receptors have been previously identified in the NCM, it remains unknown whether NPs in this brain area mediate social interactions. In songbirds, the NCM is a key site for auditory processing and song control, as are several other brain regions, including the CMM, LMAN, MMAN, Uva, and RA (Foster and Bottjer, 1998). We observed NP receptor expression in all of these areas. Specifically, we found high binding density in the NCM and RA (in European starlings), and low binding density, or binding in only a subset of individuals per species, in the CMM (all three species), LMAN and Uva (in European starlings), and MMAN (in house sparrows). These regions are components of an interconnected song control system that governs song learning and maintenance (Foster et al., 1997; Foster and Bottjer, 1998). Interestingly, we did not observe robust radioligand binding in Area X or the high vocal center (HVC), both of which constitute key sites in this network (Ziegler and Marler, 2008; Ellis and Riters, 2013). However, in house sparrows and European starlings, we observed dense NP receptor expression in the pHVC, a thin strip of cells that lines the medial edge of the HVC and lies within the margins of the NCM. Although the HVC and pHVC are neuroanatomically adjacent to each other, neural tracing studies show that the afferent and efferent projections of the pHVC are distinct from the HVC, suggesting that these two areas may be functionally distinct (Foster and Bottjer, 1998).

The social implications of NP expression in auditory and vocal brain regions remain almost wholly uninvestigated, although it is well established that NPs impact vocal behavior and learning across multiple taxa (e.g., fish: Goodson and Bass, 2000; mammals: Scattoni et al., 2008; Lukas and Wöhr, 2015; birds: Voorhuis et al., 1991; Maney et al., 1997; Goodson, 1998; Harding and Rowe, 2003; Goodson et al., 2009a; Baran et al., 2017). In the zebra finch—the most commonly used model for investigating neural control of singing behavior—pair bonding is correlated with the activation of NP receptor expression in auditory brain regions. Specifically, V1aR-like, but not OTR-like, mRNA expression is higher in the NCM and CMM of paired, relative to unpaired, females (Tomaszycki and Atchley, 2017). In combination with our results, such findings indicate that the contributions of NPs in the song control network to social grouping constitutes a fruitful potential line of research, particularly since vocalizations are likely a key driver of group formation and maintenance, at least in some avian species. For example, across different social contexts, house sparrows display distinct vocalizations, including the “flock call,” which is most readily observed in winter groups and appears to contribute to group cohesion, and a repetitive “chirrup,” which is used by both males and females to facilitate the formation of foraging groups (Elgar, 1986a,b; Anderson, 2006).

Interestingly, rock doves, but not European starlings or house sparrows, displayed high levels of binding in several brain areas that are involved in sensory pathways, including the MSt. The structural basis and functional implications of the ring-like binding pattern in the MSt of the rock doves are unclear. However, the avian MSt is known to be a heterogenous area that is composed of multiple cell types, with connectivity and neurochemical traits that differ on a mediolateral axis. Specifically, neural tracing studies implicate the medial MSt in viscerolimbic processes—which facilitate the translation of contextual stimuli into behavioral responses (Goodson and Kabelik, 2009; Kuenzel et al., 2011)—and the lateral MSt in somatosensory, visual, auditory, and motor function. We also found dense binding in additional brain nuclei—including the MVL, NIM, Bas, and DLP— that are interconnected by sensory pathways involved in transmitting visual, somatosensory, and auditory information (Atoji and Wild, 2012). Johnson and Young (2017) report that diverse taxa display NP receptors in sensory nuclei and posit that the distribution of receptors in these areas reflects “dominant socio-sensory modalities” used by each species.

### Evidence for Distinct Receptor Populations in the Avian Brain

Intraspecies comparisons of binding distributions, using multiple radioligands and binding competitors, are needed to identify heterogenous populations of NP receptors (Leung et al., 2009). However, studies that map and compare binding for both ^125^I-LVA and ^125^I-OVTA—perhaps the two most frequently used radioligands for NP receptor mapping in both mammalian and avian species—have only been conducted in two avian species: the white-throated sparrow and zebra finch (Leung et al., 2009). As described previously, Leung et al. (2009) found that ^125^I-OVTA binding was more widespread than ^125^I-LVA binding. However, the regions to which these radioligands bound were highly overlapping, leading the authors to conclude that their results could support either the presence of multiple NP receptor types, or a single receptor with differing levels of affinity for ^125^I-LVA and ^125^I-OVTA.

Although our findings regarding ^125^I-LVA and ^125^I-OVTA binding patterns are strikingly similar to Leung et al. (2009), the results from our competitive binding experiment suggest that the NP receptor populations in the LS versus the arcopallium may be composed of different receptor subtypes. Specifically, we found in sparrows and starlings that the Manning compound reduces ^125^I-LVA binding to a greater extent in the arcopallium than in the LS. Thus, our results suggest that the Manning compound affects radioligand binding in a brain region-specific manner, even in different species. However, our results also support the interpretation that the NP receptor subtypes show some degree of promiscuous radioligand binding. The binding that remains in the presence of the Manning compound may be due to radioligand binding at a different receptor subtype, likely the OTR-like receptor, VT3. If the Manning compound is indeed binding selectively to the avian V1a-like receptor, as it does primarily in mammalian systems, then our results would indicate that both radioligands bind to the V1a-like receptor, but also exhibit some affinity for the OTR-like avian NP receptor. The pharmacological cross-talk of ^125^I-LVA and ^125^I-OVTA to OTR and V1aR is already an established phenomenon which has been demonstrated in primate brain tissue (Freeman et al., 2014), and distinguishing NP receptor subtypes in primates is an active area of ongoing research. Our results support the idea that similar *in vitro* pharmacological investigations are merited in avian models as well.

### Implications for Studies Using Manning Compound as an NP Receptor Antagonist

Because of its potency as a V1aR antagonist, the Manning compound is frequently used to examine the function of specific NP receptor types in mammals, although Manning et al. (2012) caution that this compound is also a potent *in vitro* OTR antagonist and “fairly potent” *in vivo* OTR antagonist. Nonetheless, the Manning compound has also become widely used to identify the contributions of V1a-like receptors to a variety of avian social behaviors, including aggression, social attachment and affiliation, song learning, and pair maintenance behaviors (Goodson et al., 2004; Baran et al., 2016a,b, 2017).

Although the Manning compound is now a commonly used tool for determining the contributions of putative V1a-like receptors to the mediation of avian social behavior, the selectiveness of the Manning compound for specific NP receptors in the avian brain remains unknown. To the best of our knowledge, our study is the first to directly examine how the Manning compound impacts NP receptor binding in specific avian brain regions. We found that the Manning compound displaced ^125^I-OVTA more readily than ^125^I-LVA, which calls into question whether ^125^I-OVTA is labeling OTR-like NP receptors in avian brains. This finding could also indicate that the Manning compound is not specifically targeting V1a-like receptors in avian models, which merits caution for studies using it to examine V1a-like receptor functions. We suggest conservative interpretations of ^125^I-OVTA and ^125^I-LVA binding distributions, until more extensive pharmacological studies are completed with avian NP receptors. We also suggest that future avian behavioral studies using the Manning compound as an antagonist should include two treatment groups: one that combines the Manning compound with vasotocin, and one that combines it with mesotocin. This experimental paradigm has been used previously to determine if the Manning compound selectively reverses the effects of vasotocin or mesotocin on avian behavior, and to provide an added test of the hypothesis that a specific receptor subtype is predominantly involved in behavioral mediation (Goodson et al., 2004).

### Conclusion: The Value of Developing Avian Models for Social Neuroecology

Ecological conditions markedly influence social grouping across a diversity of species, but only a few studies have examined how ecological and neurobiological factors interact to mediate this behavior. Work with Amargosa pupfish (*Cyprinodon nevadensis*) indicate that social interactions are sensitive to the physical environment, and that NPs in the brain likely facilitate this ecological sensitivity. Specifically, exposure to high salinity in this species facilitates a reduction in the number of magnocellular vasotocin (VT) neurons in the preoptic area, and VT neuronal phenotypes, as well as aggression, vary with temperature regime (Lema, 2006). In addition, female meadow voles, which only form groups during winter, display same-sex affiliative behavior and group-size preference that varies with day length, temperature, and food restriction, as well as OTR binding that varies with day length (Beery et al., 2008b; Beery and Zucker, 2010; Ondrasek et al., 2015).

Similarly, European starlings, house sparrows, and rock doves display grouping behaviors that vary across ecological contexts. Notably, the behavioral profiles of each species are different in key ways that make each species advantageous for investigating particular questions about the neuroecology of collective behaviors. For example, starlings show striking seasonal patterns in flocking, such that they display high levels of aggression toward conspecifics during the breeding period, but aggregate into highly coordinated flocks that may number in the millions during the winter months (Cabe, 1993; Goodenough et al., 2017). This observation raises the question of how seasonal environmental factors, particularly day length, influence neurochemical mediation of social coordination among individual starlings. House sparrows show less striking seasonal changes in flocking than starlings; however, throughout the year, they form temporary foraging flocks that vary in size according to the divisibility of a food source, the perceived risk of predation, and distance from cover (Elgar, 1986a,b, 1987), suggesting that food availability, in relation to other environmental factors, may impact the neural mechanisms underlying flock formation. Lastly, unlike European starlings and house sparrows, rock doves are commonly found breeding in large colonies in which individuals show some degree of behavioral coordination (e.g., flushing from their nests together in response to a predator). The size, composition, and location of such colonies vary with several environmental factors, including the availability of food and nest sites (Johnston and Janiga, 1995). Thus, rock doves present an opportunity to investigate the neural mechanisms underlying colonial breeding, how these mechanisms are influenced by ecological variations, and how neuroecological regulation of colonial breeding impacts reproductive success.

To conclude, the three species examined here serve as ideal models for neuroecological research for multiple reasons: they inhabit a wide range of environments, show grouping behaviors that vary across ecological contexts, and display NP receptors in brain regions that may play a role in avian flocking. In addition, because several aspects of the NP systems are evolutionarily conserved across vertebrate taxa (Gimpl and Fahrenholz, 2001; Goodson, 2005, 2013), discoveries made using these species may guide the development of hypotheses and predictions for subsequent investigations across a much wider array of taxa.

## Author Contributions

NO and RC completed field collections of specimens. NO and SF were jointly responsible for overall experimental design and execution of the autoradiography assay. NO scored films and wrote the manuscript in conjunction with SF, with editorial suggestions from KB and RC.

## Conflict of Interest Statement

The authors declare that the research was conducted in the absence of any commercial or financial relationships that could be construed as a potential conflict of interest.
