# Supplementary material for: Nonapeptide Receptor Distributions in Promising Avian Models for the Neuroecology of Flocking
Source: Front Neurosci. 2018 Oct 16;12:713. doi: 10.3389/fnins.2018.00713 (PMC6198083; doi:10.3389/fnins.2018.00713)
Supplement: Supplementary file 1 [file Table_1.pdf]

Supplementary Material

Nonapeptide receptor distributions in promising avian models for the neuroecology of flocking

Naomi R. Ondrasek*, Sara M. Freeman, Karen L. Bales, and Rebecca M. Calisi

***Corresponding Author:** nondrasek@ucdavis.edu

1 Supplementary Data Analysis

To provide a further test of differences in ^{125}I-OVTA and ^{125}I-LVA binding, and to identify general species effects on radioligand binding patterns, we combined ^{125}I-OVTA and ^{125}I-LVA optical binding densities for all three species and performed a principal component (PC) analysis. Only the 32 brain areas showing either ^{125}I-OVTA or ^{125}I-LVA binding in at least one species were included in the analysis. The analysis produced two PCs that account for 61% of the variance in optical binding density (PC1, P<0.0001; PC2, P<0.0001; Table S1). Visual inspection of the relationship between PC1 and PC2 suggests interspecies differences in how the two components account for OBD variance (Fig. S1).

Compared to PC1 and PC2, PC3 showed considerably weaker loading across the 32 brain areas (with 20 brain areas receiving PC scores of |0.1| or less; Table S2). Thus, additional post hoc statistical tests were performed on PC1 and PC2. Two-way, full factorial ANOVAs showed significant effects of species, radioligand, and an interaction between species and radioligand on PC scores (PC1: $F_{5,48}$=126.23, P<0.0001; PC2: $F_{5,48}$=23.42, P<0.0001). Subsequent nonparametric analyses showed species differences in PC1 and PC2 scores (Steel-Dwass test, Table S3); a significant difference in PC2 scores between the ^{125}I-OVTA and ^{125}I-LVA binding conditions (Kruskall-Wallis test, Table S3); and significant differences in PC1 and PC2 scores that were associated with an interaction between species and radioligand (Steel-Dwass test, Table S3). These

outcomes support the presence of distinct ^{125}I-OVTA and ^{125}I-LVA binding patterns among house sparrows, rock doves, and starlings, although the functional significance and underlying causes of these differences, as well as instances in which significant differences were not observed, remain unclear. However, our results highlight the value of future investigations and support the use of these species as models for diverse lines of inquiry in neuroecological research.

2 Supplementary Figures and Tables

2.1 Supplementary Figures

Table S1. Percentage of variance in optical binding densities of ^{125}I-OVTA and ^{125}I-LVA that is explained by principal components.

Principal Component	Percentage of Variance
1	35.99
2	24.72
3	10.61
4	5.67
5	4.43
6	3.60
7	2.63
8	2.06
9	1.56
10	1.51
11	1.38
12	1.02
13	0.83
14	0.79
15	0.69
16	0.48
17	0.38
18	0.34
19	0.26
20	0.19
21	0.18
22	0.16
23	0.11
24	0.10
25	0.08
26	0.07
27	0.05
28	0.04
29	0.03
30	0.02
31	0.01
32	0.004

Fig. S1. Chart showing the relationship between principal components 1 and 2 for starlings (EUST), house sparrows (HOSP), and rock doves (RODO).

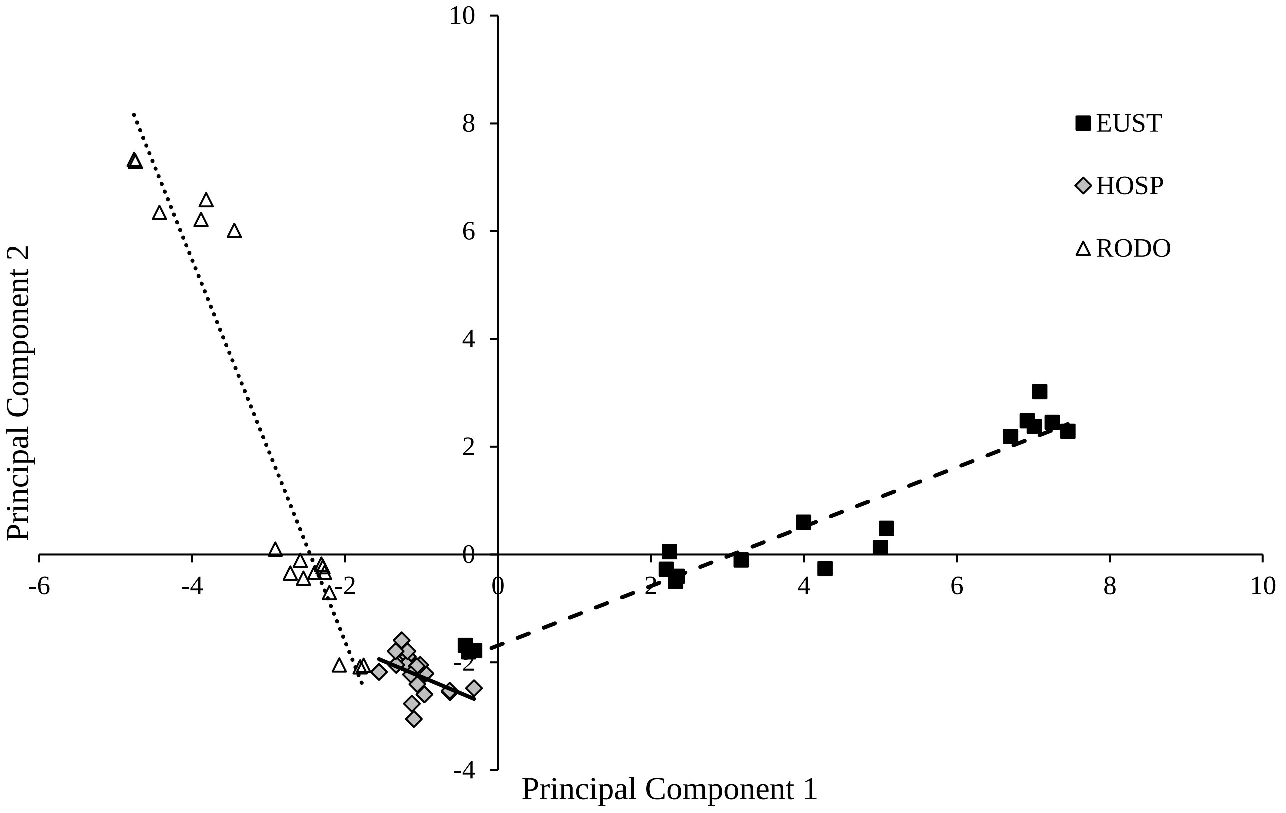

Table S2. PC loadings from an analysis of ^{125}I-OVTA and ^{125}I-LVA binding densities in house sparrows, starlings, and rock doves.

Brain Region	PC1	PC2	PC3
Ad	0.41	-0.27	0.72
Al	0.91	0.20	-0.09
APH	0.06	0.45	0.69
Bas	-0.06	0.83	0.14
CcS	0.85	0.23	-0.06
CoS	0.60	0.16	0.11
CMM	-0.52	0.49	0.12
DLP	-0.57	0.57	-0.16
DMP	-0.44	0.83	0.17
E	-0.47	0.77	0.06
H	0.57	0.57	0.23
Hp	-0.48	0.77	0.10
LHy	-0.43	0.81	0.17
LMAN	0.65	0.44	0.11
LS	0.60	0.62	-0.05
M	0.70	0.60	-0.05
MBH	0.46	0.18	-0.02
MMAN	-0.11	-0.37	0.77
MSt	-0.33	0.55	0.06
MVL	-0.46	0.80	0.13
N	0.86	0.24	-0.12
NCM	0.93	0.27	-0.09
NIM	-0.49	0.73	0.08
OMd	0.08	-0.30	0.85
OMv	0.06	-0.32	0.84
pHVC	0.90	0.19	0.20
RA	0.89	0.20	-0.03
SGP	-0.38	0.54	-0.05
TeO	0.61	0.01	0.17
TnA	0.80	0.21	-0.15
Uva	0.87	0.19	-0.01
VMH	0.72	0.20	-0.06

Table S3. Species and radioligand effects on PC1 and PC2 scores. Scores were derived from a PC analysis of optical binding densities across 32 brain regions in rock doves (RODO), house sparrow (HOSP), and European starling (EUST).

Comparison	Z statistic	*P* value
Principal Component 1		
Species Effects		
RODO vs. HOSP	5.11	**0.0045**
RODO vs. EUST	5.11	**<0.0001**
HOSP vs. EUST	5.05	**<0.0001**
Radioligand Effects		
^{125}I-LVA vs. ^{125}I-OVTA	0.57	0.57
Species*Radioligand Interaction		
^{125}I-LVA vs. ^{125}I-OVTA, in RODO	2.56	0.1071
^{125}I-LVA vs. ^{125}I-OVTA, in HOSP	1.77	0.4879
^{125}I-LVA vs. ^{125}I-OVTA, in EUST	3.53	**0.006**
Principal Component 2		
Species Effects		
RODO vs. HOSP	4.41	**<0.0001**
RODO vs. EUST	0.05	0.9988
HOSP vs. EUST	4.95	**<0.0001**
Radioligand Effects		
^{125}I-LVA vs. ^{125}I-OVTA	2.61	**0.009**
Species*Radioligand Interaction		
^{125}I-LVA vs. ^{125}I-OVTA, in RODO	3.09	**0.0245**
^{125}I-LVA vs. ^{125}I-OVTA, in HOSP	0.00	1.00
^{125}I-LVA vs. ^{125}I-OVTA, in EUST	3.09	**0.0245**
